# State and Trait Characteristics Based on Affective Temperament in Patients with Major Depressive Disorder

**DOI:** 10.1192/j.eurpsy.2025.866

**Published:** 2025-08-26

**Authors:** K. A. Kang

**Affiliations:** 1 Psychiatry, Asan Medical Center, Seoul, Korea, Republic Of

## Abstract

**Introduction:**

Affective temperament is associated with various clinical characteristics in patients with mood disorders. However, little is known about clinical characteristics based on affective temperament specifically in patients diagnosed with major depressive disorder (MDD).

**Objectives:**

This study aims to explore the impact of affective temperament on both the traits and states of individuals diagnosed with MDD.

**Methods:**

This study consecutively recruited 247 outpatients, aged 18 to 49, presenting for their initial visit to a mood disorder clinic. Affective temperament was assessed using the Temperament Evaluation of the Memphis, Pisa, Paris and San Diego-Autoquestionnaire. A Z-score of 1 or higher on each affective temperament was defined as a dominant affective temperament. The patients completed various assessments, including the Patient Health Questionnaire-9, Generalized Anxiety Disorder-7, Seasonal Pattern Assessment Questionnaire, Alcohol Use Disorders Identification Test, Hypomania Checklist-32, Interpersonal Sensitivity Measure, and Depressive Symptom Index - Suicidality Subscale. Multiple linear regression analysis was conducted to identify the impact of affective temperament on both psychiatric states and trait characteristics.

**Results:**

This study comprised 247 patients with a mean age of 29.34 ± 9.16, of whom 152 (61.5%) were female. Depressive (*β* = 0.247, *p* < 0.001) and irritable temperament (*β* = 0.138, *p* = 0.032) were positively associated with the severity of depressive symptoms, while hyperthymic temperament (*β* = -0.123, *p* = 0.041) showed a negative association. Furthermore, depressive (*β* = 0.246, *p* < 0.001), irritable (*β* = 0.195, *p* = 0.002) and cyclothymic temperament (*β* = 0.148, *p* = 0.018) were positively associated with the severity of anxiety symptoms. Cyclothymic (*β* = 0.211, *p* = 0.001) and anxious temperament (*β* = 0.136, *p* = 0.027) were positively correlated with seasonality. Hyperthymic temperament showed a positive correlation with harmful drinking patterns (*β* = 0.179, *p* = 0.006). Also, hyperthymic (*β* = 0.200, *p* = 0.002) and cyclothymic temperament (*β* = 0.140, *p* = 0.036) were positively correlated with hypomanic features. Cyclothymic (*β* = 0.255, *p* < 0.001) and anxious temperament (*β* = 0.173, *p* = 0.004) were positively correlated with hypersensitivity to interpersonal rejection. Depressive temperament (*β* = 0.184, *p* = 0.004) was positively associated with the severity of suicidality.

**Conclusions:**

Among patients with MDD, variations in psychiatric states and traits were observed based on the dominant affective temperaments. This suggests a correlation between affective temperaments and diverse psychopathological manifestations. Consequently, there appears to be a need for further research to elucidate the therapeutic implications associated with affective temperaments.

**Disclosure of Interest:**

None Declared

